# Implantable cardioverter-defibrillators in non-ischaemic cardiomyopathy: a need or not?

**DOI:** 10.1007/s12471-023-01765-4

**Published:** 2023-02-20

**Authors:** Joris R. de Groot

**Affiliations:** Heart Centre, Department of Cardiology, Amsterdam University Medical Centres, University of Amsterdam, Amsterdam, The Netherlands

In the Netherlands, sudden cardiac death (SCD), usually caused by ventricular fibrillation, remains a major cause of mortality, with approximately 8000 cardiac arrests taking place outside the hospital annually (www.hartstichting.nl/hart-en-vaatziekten/cijfers-hart-en-vaatziekten). The majority of cases of SCD occur in subjects with an *a priori* low risk, as they outnumber the high-risk subjects in whom risk stratification and primary prevention of sudden death can be instituted [1]. Hence, aside from training lay public in basic life support and increasing access to public automatic defibrillators, our efforts in practice are aimed at SCD prevention in high-risk patients. Indeed, large randomised clinical trials have demonstrated that mortality can be reduced with implantable cardioverter-defibrillators (ICDs) in patients with a diminished left ventricular ejection fraction, both in primary and secondary prevention settings [2, 3]. In the Netherlands, the increased uptake of ICDs has subsequently reduced the chance of recording a shockable rhythm at cardiac resuscitation when compared with the pre-ICD era, indicating that a proportion of high-risk patients with an ICD are withdrawn from the group that is resuscitated [4]. Consequently, the guidelines of the European Society of Cardiology recommend implantation of an ICD in patients with ischaemic cardiomyopathy and a left ventricular ejection fraction (LVEF) of ≤ 35% despite at least 3 months of optimal medical heart failure therapy and a New York Heart Association (NYHA) class II or III, (class I, level of evidence A), and state that ICD implantation should be considered in patients with non-ischaemic cardiomyopathy, an LVEF of ≤ 35% despite optimal medical therapy and a NYHA class II or III (class IIA, level of evidence A) [5]. The latter recommendation represents a downgrade in recommendation compared with the 2015 recommendations, and this has been fuelled mostly by the DANISH study, which showed a trend in 1116 patients with non-ischaemic cardiomyopathy randomised to ICD or to no ICD after optimal medical therapy. ICD therapy in the DANISH study caused a significant reduction of SCD, but not in all-cause mortality (hazard ratio [HR] 0.87; 95% confidence interval [95% CI] 0.68–1.12; *P* = 0.28) [6]. Thus, there is discussion about the need for ICD therapy in patients with non-ischaemic cardiomyopathy in the modern era of advance heart failure medical therapy.

In this issue of the Netherlands Heart Journal, Theuns et al. present a meta-analysis of the available randomised evidence for ICD therapy in patients with non-ischaemic cardiomyopathy [7]. They include 6 randomised controlled trials and 2 non-randomised clinical trials. The majority of trials randomising between optimal medical therapy and ICD derive from the early years of this century, and three of them (CAT, AMIOVERT and DEFINITE, published between 2002 and 2004) were moderately sized with between 103 and 458 patients per trial. The COMPANION and SCD-HeFT trial (published in 2004 and 2005), however, were large intervention trials, but included both patients with ischaemic and non-ischaemic cardiomyopathy [2, 8]. The DANISH study, published in 2016, is, therefore, the only contemporary trial on ICD therapy in non-ischaemic cardiomyopathy patients available.

Theuns et al. meta-analysed the RCTs and report that ICD therapy reduced the risk of all-cause death by 24% compared with medical therapy (HR 0.76, 0.62–0.93, *p* = 0.008), with more benefit in trials with follow-up of < 3 years compared with ≥ 3 years. There was no difference in the outcomes in the medical therapy group when stratified for use of amiodarone. The comparison with non-randomised studies supports these findings: when pooling the data of the 6 RCTs with the EU-CERT-ICD, the HR for death was 0.72, (95% CI 0.60–0.87, *p* < 0.001).

When the use of biventricular pacing is taken into account, the picture becomes less clear: the COMPANION trial compared cardiac resynchronisation therapy with defibrillator (CRT-D) with medical therapy and showed a reduction in all-cause mortality of 50% (HR 0.50, 95% CI 0.29–0.88, *p* = 0.015). On the other hand, the DANISH study compared CRT‑D with CRT *without* defibrillator (CRT-P), and failed to show a reduction in all-cause death (HR 0.91, 95% CI 0.64–1.29, *p* = 0.59). The pooled analysis showed no reduction in all-cause mortality (HR 0.74, 95% CI 0.47–1.16, *p* = 0.19). Interestingly, the recently published sub-analysis of COMPANION, comparing patients receiving CRT‑D with CRT‑P, confirms the findings of the original trial (HR 0.54, 95% CI 0.34–0.86), suggesting that the difference between COMPANION and DANISH relates—at least in part—to a lower risk of mortality due to more effective contemporary heart failure management [9].

To translate these finding to the situation in the Netherlands, Theuns et al. estimated the effect of ICD therapy in patients with non-ischaemic cardiomyopathy enrolled in the Dutch DO-IT trial. Using the numbers from their meta-analysis, they calculated that the absolute risk reduction of ICD therapy compared with medical therapy amounts to 3.7%, and that the number needed to treat consequently is 27.

The practice of ICD implantation has grown over the last 10–15 years to approximately 6000 implantations every year in the Netherlands alone, with obvious repercussions on healthcare budgets and considerable rates of device-related complications. There is pressure from regulating bodies to reduce the number of ICD implantations, particularly in patients with non-ischaemic cardiomyopathy. The contribution of Theuns et al. shows that ICDs reduce all-cause mortality in these patients, with a number needed to treat of 27. Consistently, the Dutch DO-IT registry (39% non-ischaemic cardiomyopathy), aimed at building prediction models for appropriate ICD therapy, showed a similar appropriate ICD therapy rate of 3.2% per year [10]. The story is less clear regarding the use of defibrillators in addition to CRT, as COMPANION and DANISH show conflicting evidence. Whether this means that ICD therapy is not indicated in resynchronisation candidates with non-ischaemic cardiomyopathy, has not been resolved. The current evidence base is not very broad and prospective clinical registries as well as randomised trials with contemporary heart failure management are needed to shed more light on this matter.
